# Oral-care adherence. Service design for nursing homes – initial caregiver reactions and socio-economic analysis

**DOI:** 10.3205/000306

**Published:** 2022-03-31

**Authors:** Stefan Wagner, Ingrid Rosian-Schikuta, Jorge Cabral

**Affiliations:** 1 Aarhus University, Aarhus, Denmark; 2 Gesundheit Österreich GmbH, Vienna, Austria; 3 University of Minho, Braga, Portugal

**Keywords:** oral hygiene, toothbrushing compliance, self-care, service design, telemonitoring, electric toothbrushes, sensors, socio-economic analysis

## Abstract

**Background:** Lack of proper oral care among elderly people in nursing homes is associated with increased morbidity and hospitalisation. The ability of the individual to maintain sufficient oral self-care is difficult for caregivers to assess, and thus, caregivers often risk providing suboptimal oral care. Sensor-based tools exist that can support the caregiving staff in achieving a better understanding of who among the elderly are able to perform proper self-care, and who cannot and thus need additional assistance from caregivers. How such systems should be designed and deployed in nursing homes, and how they will be perceived by caregivers has not been investigated sufficiently yet.

**Objectives:** The aim of this study was to gain a better understanding of how caregiving staff perceives the introduction of sensor-based systems that allow the caregivers to automatically discover who among the elderly residents are able to adhere to the given recommendation on daily oral self-care, and who among them are in need of further assistance, as well as whether there is potential for saving costs.

**Methods:** In a mixed methods qualitative study, we visited three nursing homes where we had recently deployed, or were about to deploy, electrical toothbrushes and a basic oral-care adherence aid system. Nursing home staff was interviewed during the field studies about their initial reactions to introducing such a system as part of their daily workflow. The field study was supported by a literature review.

**Results:** Caregiving staff welcomed the introduction of a sensor-based oral-care adherence aid system, which would identify any elderly who could no longer achieve a sufficient level of oral self-care.

Improving oral care for the elderly may not only prevent serious consequential diseases, but also generate considerable savings with a return on investment of at least 1:2.5.

**Conclusion:** Sensor-based oral-care adherence aid systems that monitor oral-care adherence, meaning the ability of the individual elderly to properly perform teeth and/or denture brushing as part of normal self-care efforts, appear useful and relevant to introduce. More work is needed to provide a better understanding of the long-term user experience of both caregiving staff and elderly. There is also a need for more high-quality long-term clinical studies of further preventive effects of oral hygiene measures and their economic benefit.

## Zusammenfassung

**Hintergrund:** Mangelnde Mundhygiene bei älteren Menschen in Pflegeheimen ist mit erhöhter Morbidität und Krankenhausaufenthalten verbunden. Für das Pflegepersonal ist es schwer, einzuschätzen, ob ein Pflegeheimbewohner eine ausreichende Mund- und Zahnpflege erhalten hat oder nicht, und es besteht die Gefahr einer suboptimalen Mundhygiene. Sensorbasierte Technologien können das Pflegepersonal dabei unterstützen, zu bestimmen, welche älteren Menschen in der Lage sind, ihre Mundhygiene selbst ordnungsgemäß durchzuführen und welche nicht, und daher zusätzliche Unterstützung durch das Pflegepersonal benötigen. Wie diese sensorbasierten Technologien designt werden, wie sie in Pflegeheimen angewendet und vom Pflegepersonal ange-nommen werden, ist noch nicht ausreichend untersucht.

**Zielsetzung:** Ziel dieser Studie war es, einen besseren Einblick zu gewinnen, wie das Pflegepersonal die Einführung von sensorbasierten Technologien annimmt, die es dem Pflegepersonal ermöglichen, automatisch zu erkennen, welche Pflegeheimbewohner in der Lage sind, ihre tägliche Mundhygiene selbst durchzuführen, und welche weitere Unterstützung benötigen. Außerdem wurde abgeschätzt, ob Kosteneinsparungen möglich sind.

**Methodik:** Es wurden qualitative Feldstudien durchgeführt. Wir besuchten drei Pflegeheime, wo wir elektrische Zahnbürsten und ein sensorbasiertes System zur Unterstützung der Mundhygiene einsetzten. Das Pflegeheimpersonal wurde während der Feldstudien zu ihren ersten Reaktionen auf die Einführung eines solchen Systems als Teil ihres täglichen Arbeitsablaufs befragt. Eine systematische Literaturrecherche ergänzte die Feldstudie.

**Ergebnisse:** Das Pflegepersonal begrüßte die Einführung eines sensorbasierten Mundhygiene-Adherence-Systems, mit dem ältere Menschen identifiziert werden können, die selbst kein ausreichendes Niveau der oralen Mundhygiene erreichen. Die Verbesserung der Mundhygiene bei älteren Menschen kann nicht nur schwerwiegende Folgeerkrankungen verhindern, sondern auch erhebliche Einsparungen mit einem Return on Investment von mindestens 1:2,5 erzielen.

**Schlussfolgerung:** Die Einführung sensorbasierter Systeme zur Unterstützung und zum Monitoring der Adherence bei der Mundpflege – also der Fähigkeit älterer Menschen, Zähne- und/oder Prothesenputzen als Teil der normalen Selbstpflege ordnungsgemäß durchzuführen –, erscheinen nützlich und relevant. Es sind weitere Studien angezeigt, um ein besseres Verständnis der langfristigen Nutzererfahrungen sowohl des Pflegepersonals als auch der älteren Menschen zu erhalten. Darüber hinaus besteht Bedarf an qualitativ hochwertigen klinischen Langzeitstudien zu weiteren präventiven Effekten von Mundhygienemaßnahmen und derem wirtschaftlichem Nutzen.

## 1 Introduction

European national public healthcare systems are facing massive economic and caregiver-manpower challenges due to changes in the demographic composition of the population, including a significant increase in the number of elderly in public nursing homes, which is expected to double until 2045 [1]. To mitigate these challenges, ambient assisted living and pervasive healthcare service design tools are being developed for empowering the elderly to be more independent and self-sufficient [2]. This is expected to reduce the pressure on caregiving staff and optimise to whom manual care should be provided [3].

One important self-care ability is oral care. It is essential to maintain it at an adequate level and on a daily basis. Suboptimal oral-care in the elderly is associated with increased morbidity and mortality [4], [5].

A Swedish study found that up to 77% of elderly nursing home residents were in need of oral care, while only 7% of these received sufficient oral care [6]. Maintaining oral care is often impeded by the residents’ disabilities, and associated challenges including co-operation, communication, and access, which implies that the offered oral care services need to address these challenges [7], [8].

Sensor-based tools can support the caregiving staff to achieve a better understanding of which elderly are able to perform proper self-care, and which who cannot, thus requiring additional assistance from caregivers [9]. However, how such systems should be designed and deployed in nursing homes has not been investigated yet.

Oral care is a challenge in nursing homes, specifically the ability of a person to perform sufficient oral self-care, either autonomously, or with the aid (full or partial) of a caregiver, a concept also referred to as ‘adherence’ or ‘compliance’ when used in a clinical context, more specifically called ‘oral-care adherence’ or ‘oral-care compliance’ [9]. We may define this concept as follows: “oral care adherence is the ability of the individual person to achieve proper oral self-care, either autonomously or assisted by a caregiver, to provide oral care on a daily basis, in compliance with the general recommendations given by national and international dentistry organisations and societies and/or a specific regimen of oral self-care prescribed by a dentist or other health professionals”. The general recommendation in the field is: two brushings per day of a two-minute duration each [10], [11].

Oral care is very important to maintain, and the lack of proper oral care in the elderly is associated with increased morbidity and hospitalisation, due to respiratory and other diseases, and increased mortality [4], [5], [12], [13], [14].

A major challenge in caregiving is the more or less sudden changes in the ability of the individual elderly to do oral self-care, especially if the lack of proper self-care has gone unnoticed for a prolonged period of time, after which the damage to the mouth and teeth may cause severe pain and lead to irrevocable damage to the teeth and gums in the oral cavity because of difficulties in dental treatment of such physically and possibly mentally weak elderly persons. Furthermore, poor oral hygiene may lead to other diseases, spreading from the mouth area, including heart and lung infections [12], [13], [14].

Pervasive health products may support caregiving staff to achieve a better understanding of which elderly are able to perform proper self-care, and who cannot and thus would need additional assistance from caregivers. One such experimental tool, CARIOT BRUSH, has been developed by a group of universities, end-user organizations, and companies, which specifically include the ability to monitor the level of oral self-care adherence of its users [15]. Based on the CARIOT BRUSH platform [15] combined with the CARIOT citizen overview triage tool [7] and an Oral-B electrical toothbrush [16], this care-service tool can effectively and accurately monitor all brushing actions of elderly citizens in nursing homes and home-care scenarios.

One coping strategy for healthcare systems is to rely on their citizens’ ability to self-care for as long as possible, in order to reduce strain on caregiving staff. Self-care is defined by the US National Library of Health as the “[p]erformance of activities or tasks traditionally performed by professional health care providers. The concept includes care of oneself or one’s family and friends” [17]. Thus, self-care is a widely used concept that has been employed to describe patients performing self-medication, self-rehabilitation, and/or self-measurements [9].

The individual person’s (often referred to as a patient or citizen) ability to follow prescribed guidelines and treatment plans is covered by the concept of ‘adherence’. The level of adherence relates to the degree to which a patient correctly follows given clinical advice [9]. Adherence is defined as “the process in which a person follows rules, guidelines, or standards, especially as a patient follows a prescription and recommendations for a regimen of care” [18], or as “the extent to which the patient continues the agreed upon mode of treatment under limited supervision when faced with conflicting demands, as distinguished from compliance or maintenance [19]. Many alternative definitions of adherence exist [9].

A well-known example is medical adherence, where the ability of the individual to adhere to and comply with the prescribed medicine regimen of care is important in order to avoid resulting morbidity or hospitalisation, and even early death. Also, self-training or self-exercise adherence has attracted attention in recent years, as it has been shown that a sufficient level of self-exercise is a main element in keeping elderly citizens healthy and mobile [9].

Interventions, measures, and means to increase adherence fall under the category of disease management relying on the focused application of resources in order to improve healthcare processes [9]. Wagner et al. have previously introduced the concept of ‘adherence strategies’ and ‘adherence strategy engineering’, where they define an ‘adherence strategy’ as the “means, measures, and interventions used to facilitate patient adherence during a healthcare process as part of the overall disease management” [9].

Adherence strategies are further divided into ‘adherence verifiers’ which are “defined as elements that quantify the adherence levels of a given healthcare process and the resulting data quality of the healthcare process” [9]. The automatic registration of tooth brushing actions is an example of such an adherence verifier. Next, ‘adherence aids’ are defined as “tools and technologies that will help the patient to better adhere to a prescribed treatment plan by providing passive or active guidance” [9]. Thus, the CARIOT citizen overview triage system can be seen as an adherence aid that provides support for the caregivers, but not as a tool for supporting the citizens to perform self-care.

A further relevant concept includes ‘service design’, which “can provide tools for addressing challenges related to the adoption of pervasive health technologies” [20]. Thus, service design as a concept is related to adherence strategy engineering, as it aims to use the “digitalization of healthcare for supporting patients to have a stronger role in their care” [20].

In other words, both adherence strategy engineering and service design aim at providing shared decision-making tools for empowering patients in care-related decisions, combined with sensor devices for helping patients in building self-awareness of their health, and digital platforms for providing new ways for patients to communicate with their peers and care professionals.

Thus, service design “offers tools for harnessing these new digital enablers for patient empowerment through understanding ecosystems, user journeys and value co-creation models needed for successful implementation and adoption in context of care” [20].

As is true for most types of service tools, the initial acceptance of a new care service design tool by caregiving staff and the elderly residents themselves is key to a successful adoption [9].

To the best of our knowledge, no prior work has studied the initial acceptance of caregiving staff on using an adherence aid tool for monitoring oral-care adherence and providing an automated early warning system. Also, no work has studied the economic implications of introducing such a tool in the clinical setting.

A range of personal care tools and devices does exist, mainly smartphone app-based tools, but these are not useful in a community care setting, such as a nursing home and similar care institutions and facilities, as they are designed for private home usage.

Thus, the aim of this study was to gain a better understanding of how caregiving staff perceives the introduction of a sensor-based system that allows the caregivers to automatically triage which elderly residents are able to adhere to the given recommendation on daily oral self-care and which elderly are in need of further assistance from caregivers. A further aim of the study was to understand the economic implications of introducing these technologies in a public long-term care facility.

## 2 Methods

### 2.1 Technology platform

An open-source citizen monitoring and adherence aid system developed by a group of universities, end-user organisations, and telecare companies (ALIVIATE, Denmark) was adapted to include an overview of individual citizen oral-care adherence, including both oral self-care and caregiver-provided care.

An electrical toothbrush by Oral-B was adapted to allow for automatic registration of all brushing actions with the CARIOT gateway, a multi-purpose medico gateway that can collect data from multiple medico and telecare devices. The electrical toothbrush measures the duration and frequency of brushings only. Thus, the quality of the brushings is not evaluated and cannot be guaranteed.

**Figure 1:**
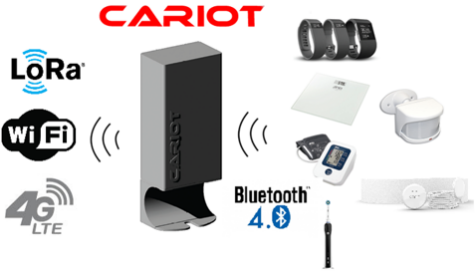
The CARIOT homecare sensing platform (center of the image) communicates with an Oral-B electrical toothbrush and sends the data via a range of different communication protocol standards, Lora, WiFi or 4G to the automatic adherence aid system.

**Figure 2:**
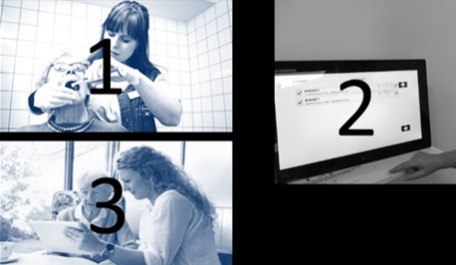
1) The elderly residents themselves or a caregiver performs the brushing. Data are automatically registered and sent to the adherence aid systems database. 2) The Triage Citizen Overview tool – a wall-mounted touch-screen computer unit – shows the current status of the brushing actions. 3) Caregivers, citizens, and/or relatives can jointly review historic brushing action statistics on a tablet or web client.

**Figure 3:**
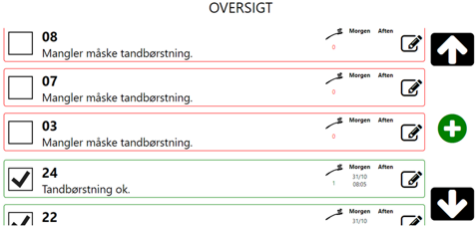
The CARIOT Triage Citizen Overview Screen. The original Danish text translates into English as follows: “Oversigt” is “Overview”, and “Mangler måske tandbørstning” is “May need toothbrushing”, and “Tandbørstning ok” is “Toothbrushing is OK”. In the columns to the right, “Morgen” is “Morning”, and “Aften” is “Evening”. Here, citizens’ names are shown anonymised (as 03, 07, 08, 22, 24) and their current brushing actions are shown. Red means no brushing done today, and green means brushing was performed as prescribed by the dentist. This screen is placed at the caregiver’s office.

The system is illustrated in Figure 1 (Fig. 1), Figure 2 (Fig. 2) and Figure 3 (Fig. 3). In Figure 1 (Fig. 1), the CARIOT home care gateway is shown, along with its communication interfaces. The home gateway can connect using Lora (WAN), WiFi or 4G. Locally, it communicates with most devices using either Bluetooth Low Energy (BLE), but can also use both Lora and WiFi radio communication to connect to local medico devices. In Figure 2 (Fig. 2), the connection between the electrical toothbrush and the adherence aid system is shown as the three typical workflow processes at a nursing home. Figure 3 (Fig. 3) showcases sample anonymised data on the CARIOT adherence aid system overview screen.

### 2.2 Study design

A mixed methods qualitative study methodology was adopted. This included field studies using contextual inquiry where we observed and asked caregiving staff informal questions about their initial reactions to the introduction of such a system, combined with semi-structured interviews with caregiving staff.

During the contextual inquiry, we followed caregiving staff at the nursing home, asking informal questions in order to gain new knowledge. Relevant information was noted on paper and later transcribed. Next, the semi-structured interviews were performed using a pre-filled paper form as the structured part of the interview, where we presented the prepared questions and noted the answers. Next, we allowed for any additional non-structured information originating from the caregivers, which was also noted on the paper form.

Finally, after the project ended, we interviewed caregiving staff during two group meetings, which were recorded and transcribed.

#### 2.2.1 Literature review

In addition, a systematic literature search was conducted, identifying available evidence on the effectiveness and economic benefits of oral care for elderly people in nursing homes, with the aim to gather evidence for calculation of potential economic consequences based on literature search. The following questions for the literature search were addressed:

1) What evidence is available for the effectiveness of oral health care prevention measures for elderly people in nursing homes?

2) What costs were reported for prevention measures for elderly people in nursing homes?

#### 2.2.2 Search

The international database MEDLINE was searched via Ovid (on 14^th^ March 2019) linking different search terms regarding different terms of oral health (oral hygiene measure, oral health, tooth brushing, dental care), the population (elderly people, nurse, geriatric), the setting (homes for aged, nursing home, nursing care or home nursing, retirement home, old people home) and economic terms (costs, cost analysis, economic or economic evaluation or cost effectiveness), utilising both relevant MESH-terms and text words. The search was limited to literature from 2008 to 2019, the electronic search was supported by a hand search (Google Scholar, clinical trial register (https://clinicaltrials.gov), www.nice.org) done in March 2019.

#### 2.2.3 Eligibility

Studies were considered eligible for inclusion if they met the following pre-defined eligibility criteria:

population: interventions targeting residents in nursing homes of any sex; interventions: oral care interventions (e.g. tooth bushing, cleaning of dentures, cleaning of tongue, with or without information communication (ICT) support; control: usual or no oral care; outcome: mortality, morbidity, health-related quality of life (QALY), patient satisfaction, any reported costs/benefits. Furthermore, eligible for inclusion were studies written in English and German; inclusion of study type for effectiveness studies were restricted to systematic reviews and meta-analysis of randomised controlled trials (RCT) and in which risk of bias of individual studies have been assessed; for economic studies/evaluations, no restrictions on study type or quality assessments have been applied.

#### 2.2.4 Study selection

Identified studies were imported in EndNote and duplicates removed. Abstracts and titles, as well as the full text selection, were screened by two reviewers.

#### 2.2.5 Data synthesis/study quality

**Figure 4:**
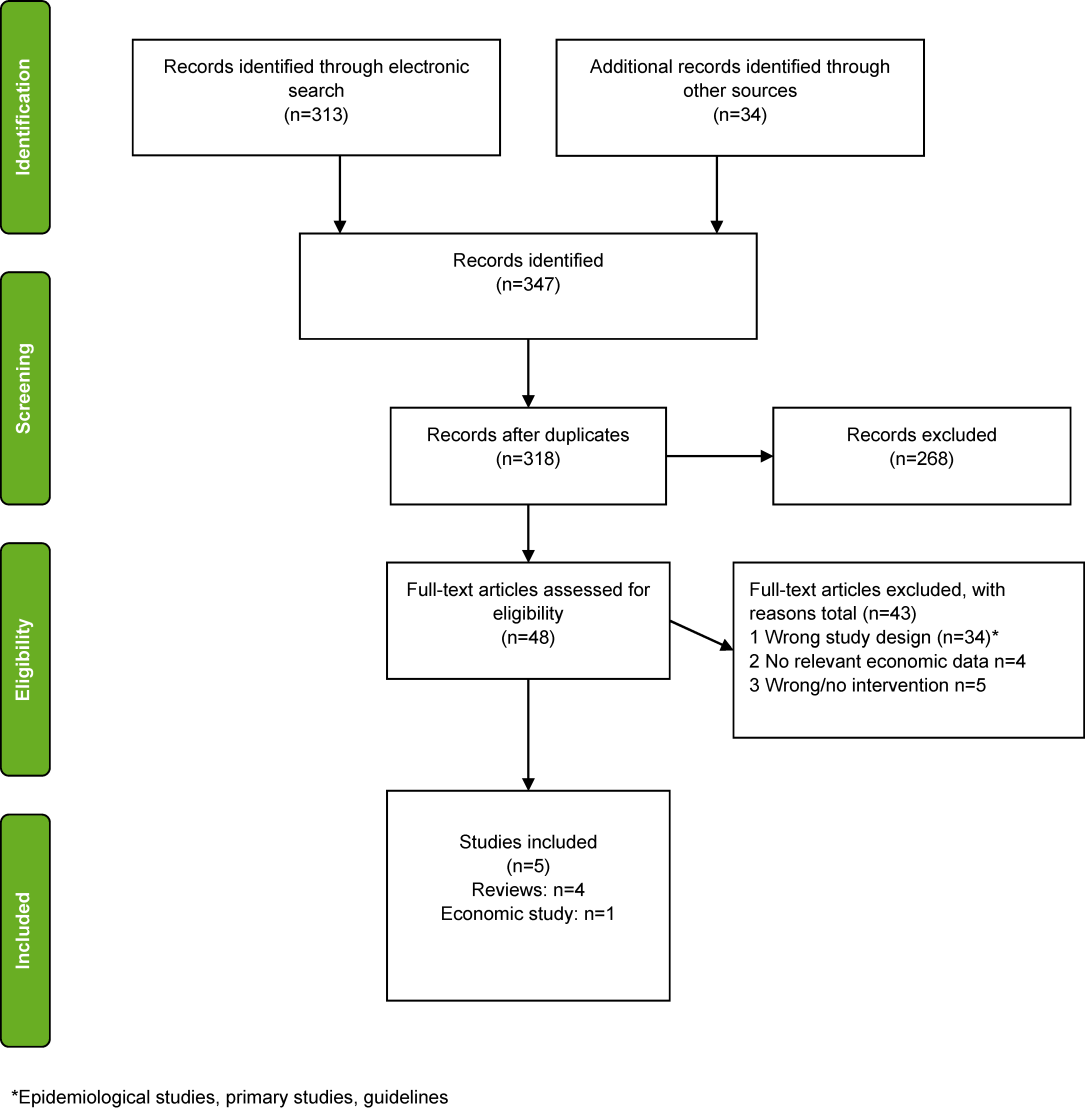
Search and selection process, PRISMA Flow Diagram

The search and selection process is presented with a PRISMA flow diagram (Figure 4 (Fig. 4)). Due to resource constraints, data synthesis was done narratively. Information relevant for answering the research questions have been extracted; a quality assessment of the included systematic reviews or meta-analysis have not been conducted; in particular because systematic reviews and meta-analysis were only deemded eligible for inclusion if a quality assessment of the included studies was already performed. Cost data for the ‘help me’ brush system were provided by the consortium of the pilot project.

### 2.3 Participants

Participants were recruited from three typical Danish public sector nursing homes in three Danish municipalities, either before or during deployment of the electrical toothbrushes and the CARIOT Brush oral-care adherence adherence aid system. In total, we followed, observed, and informally interviewed 20 caregivers, while deploying the system in the homes of 25 elderlies.

## 3 Results

### 3.1 Field study

A range of themes emerged from the study. In the following, we provide relevant supporting examples.

In general, the caregivers and the elderly residents had a positive attitude towards the electrical brushes and gateways. First, during the contextual inquiry field studies, all observed and confronted caregivers informally prompted that they found it relevant to introduce an oral-care adherence aid system to support their work and ensure high quality oral care. Thus, the following selected comments were recorded: “I think this is a great idea, we should definitely have such automatic registration equipment here” (Nurse 1); “If it can help me avoid errors and forgetting brushings, I think this is definitely a good thing, why wouldn’t it be?” (Caregiver 1). “I think this sounds like a really good idea” (Caregiver 2). “This is definitely a relevant tool to have for our planning” (Caregiver 3). “I find it absolutely brilliant if this small black box (pointing to the CARIOT gateway) can automatically register all brushing efforts, really good” (Caregiver 4). “Electrical toothbrushes that automatically remind us to brush the teeth of the elderly if it’s forgotten, I think that is a very good idea” (Caregiver 5). Several similar comments were recorded from six other caregivers.

Also, caregivers were generally aware that daily and high-quality oral care was very important: “Oral care is just as important as changing a diaper or preventing bed-soars (which are pressure wounds due to the elderly lying too long in the same position)” (Caregiver 6). “It is a basic part of our everyday morning routine we should never forget to do it” (Caregiver 7).

Several caregivers provided comments concerning that they in fact did not forget to provide oral care, but that the frequent use of “substitute” workers, unfamiliar with the usual schedule and residents “could benefit highly” from the technology. Relevant comments include: “I personally never forget to provide oral care, but sometimes a substitute may not be aware that they should do the brushing, and it is forgotten” (Caregiver 8), a statement repeated by four other caregivers in similar comments.

Some caregivers were mildly skeptical about the introduction of electrical toothbrushes. “They make a lot of noise, I don’t think they will allow me to brush using this one” (Caregiver 9). “Do they really need to be so loud? I think we will scare her [explanation: referring to an elderly resident]” (Caregiver 10). “Good luck with trying to get him to use that monster [explanation: referring to another male resident] (Caregiver 11). However, the majority did not comment on the noise levels made by the electrical brushes.

The triage screen was found positive by the caregiving staff. “I think this is a really useful tool” (Caregiver 12). “It seems easy enough to understand. You just look at the screen, and see that this person missed his brushing, and then you can get it done” (Caregiver 13).

Also, there was some scepticism raised towards the technology: “I think it will be OK, but really, we don’t want a lot of different systems around, it can become very confusing” (Caregiver 14). “OK, as long as it’s just that little box that plugs into the wall-power, I guess its fine, but please, I don’t want to do any more manual registrations, OK?” (Caregiver 15). “What happens if they get wet? Do they break? [note: referring to the CARIOT gateways]” (Caregiver 16).

Finally, the caregivers that tried the CARIOT citizen overview screen provided some early insights as well: “I think it’s quite easy to get a quick overview of who actually had their teeth brushed already, and who we forgot; I think this is a very usable tool in the future” (Caregiver 17).

### 3.2 Literature review

The number of studies identified, reviewed and selected and the reasons for exclusion are summarised in the PRISMA flow diagram (Figure 4 (Fig. 4)).

In total, five studies could be retrieved for analysis. Four systematic reviews and meta-analyses [4], [5], [21], [22] for answering effectiveness (question 1), one study [23] for economic issues (question 2).

Only studies for the evaluation of the effects of good oral health in association with pneumonia for elderly people in nursing home could be identified; no systematic reviews or meta-analysis e.g. regarding the association between good oral health and cardiovascular diseases, diabetes or other diseases could be identified. From the overall pool of 318 studies, no systematic review could be identified that assessed ICT monitoring of tooth brushing in nursing homes.

All four systematic reviews and meta-analysis [4], [5], [21], [22] reported a statistically significant reduction of pneumonia-associated mortality due to professional oral care versus usual care for elderly people in nursing homes or hospitals. In the meta-analysis of Sjogren et al. [5], effects of oral care on mortality have been assessed, however not the effects on pneumonia.

In the systematic review of Sjogren et al. [4], the authors found that approximately one in ten deaths and that at least 10% of hospitalisations due to pneumonia and other respiratory diseases are preventable. Regarding the effects of oral care on the prevention of pneumonia, the four included RCT in the review of Sjorgren et al. [4] yielded an absolute risk reduction in the range from 6.6 to 11.7%.

In the Cochrane meta-analyses (Liu et al. [22]), the authors were unable to determine whether professional oral care resulted in a lower number of first episodes of pneumonia compared with usual care over a 24-month follow-up (RR 0.61, 95% CI 0.37 to 1.01, based on one RCT (low-quality evidence)). Whereas meta-analysis in Kaneoka et al. [21] reported a statistically significant risk reduction of getting pneumonia due to mechanical oral care (RR 0.61, CI 0.40 to 0.92), meaning a risk reduction of about 40%. The results of Liu et al. [22] were based on one RCT, while those of Kaneoka et al. [21] were based on three RCT, which may be the reason for the different results of effects. However, the results of Kaneoka et al. [21] are consistent with the findings of Sjogen et al. [4].

One study (Costa et al. [23]) could be retrieved to identify economic data. The authors analysed additional costs of pneumonia in nursing home residents in France. They assessed direct costs for treatment of pneumonia from 13 nursing homes. The main findings are that 18% experienced at least one episode of pneumonia during the 1-year follow-up. Additional costs because of pneumonia were 2,813.00 EUR per resident and year. This could be saved if pneumonia were avoided.

### 3.3 Estimation of economic consequences

Based on available data from the literature search, the savings potential would be 2,813 EUR/resident and year, of which 18% are statistically likely to catch pneumonia [23], of which about 40% may be avoided [4], [21]. Thus, the savings potential would be 2,813*0.18*0.40=202.00 EUR/year and resident. Comparing the costs of the monitoring gateway with 80.00 EUR per resident and year with the savings potential yields a return on investment of 1:2.5. This is a rough estimate taking only the savings of the prevention of pneumonia into account. It could be even higher, since other diseases that could be prevented as well are not included here. Also, mortality has not been assessed. The calculations have some limitations. They are only based on the costs of pneumonia from France. In other countries, the costs of pneumonia may be different (e.g. the Danish study suggested that the costs of pneumonia in elderly is 17,333.00 EUR per hospitalisation on average. There may be different incidence rates in nursing homes in other regions; and the size of the risk reduction of oral care, which should be based on high-quality primary studies, could influence the results.

## 4 Discussion

In general, caregivers did find the introduction of electrical toothbrushes and a basic oral-care adherence aid system as relevant, although there were fears that the noise levels of the electrical toothbrushes would frighten some of the weaker elderly users.

Also, caregivers were generally aware that daily and high-quality oral care is very important, and recognised that they were actually not able to tell the current status of their citizens, a problem that they said was related with the frequent use of ‘substitute’ workers, unfamiliar with the usual schedule and residents.

Specifically, they welcomed the introduction of an oral-care adherence aid system to support their work and ensure high-quality oral care. The simplicity of the solution, including the ‘plug and play’ functionality, and the simple triage citizen overview screen were received positively by the members of the caregiving staff.

Finally, some caregivers were mildly skeptical about the introduction of electrical toothbrushes, especially the noise levels, and in general concerning the technology, which they did not want to be overly complex to use.

Thus, the introduction of adherence aid systems for oral care (and other care activities) appears relevant to further investigate as a potential ‘service design tool’, including support for ‘shared decision-making tools’ which can empower caregivers and patients in care-related decisions, and the electrical toothbrushes as care sensor devices which could help patients in building self-awareness of their health.

The present solution does not support ‘patient empowerment’, as reminders are provided to caregiving staff only. Thus, in order for the system to support ‘patient empowerment’, additional user interface elements are needed as part of the service design. This could include localised in-home light and sound reminders. To the best of our knowledge, no previous studies have looked into staff acceptance of introducing an oral-care adherence aid system in nursing homes. Related studies in nursing home care settings have only studied the ability and willingness of caregivers to provide care, not their reactions toward an automated adherence aid system [22], [23]. Likewise, no full socio-economic studies have been performed on the efficiency and effectiveness of oral-care adherence aid systems. Thus, high-quality and long-term studies are needed to gain more accurate socio-economic data.

Future work includes: providing statistics on the actual use of the toothbrushes in terms of frequency and duration of the individual brushings in order to provide insights into the actual adherence level of the individuals. Also, qualitative interviews should be performed with both caregiving staff and the elderly residents themselves on how they perceive such an intervention during a prolonged period of study. Finally, it would be interesting to experiment with different types of adherence strategies, e.g. ranging from simple adherence verifiers (like introducing the triage overview system) to various types of adherence aids, both for the caregivers and for the citizens themselves. These could include localized reminders and nudging elements, using light and/or sound, as well as text-based reminders and notifications sent to smart phones, tablets, and even the wall-mounted touchscreen unit.

## 5 Conclusion

Sensor-based oral-care adherence aid systems that monitors the oral-care adherence, meaning the ability of the individual elderly to properly perform teeth and/or denture brushing as part of normal self-care efforts, appear useful and relevant to introduce. On the one hand, to prevent serious subsequent diseases caused by inadequate oral hygiene and, on the other hand, to realise the potential savings. More work is needed in order to provide a better understanding of the long-term user experience of both caregiving staff and elderly. There is also a need for more high-quality long-term clinical studies of further preventive effects of oral health measures and their economic consequences.

## Notes

### Funding

Under the project number AAL-2018-5-184 and AAL-2020-7-239 this work was supported by the Active and Assisted Living Programme (AAL Programme) and by grants of the Danish Agency for Science, Technology and Innovation, and Innovation Fund Denmark as part of the HELP ME BRUSH and ORASTAR research projects.

### Competing interests

The authors declare that they have no competing interests.
